# Error Detection and Correction in Chinese Radiology Reports Using Large Language Models: Real-World Clinical Validation Study

**DOI:** 10.2196/94689

**Published:** 2026-08-21

**Authors:** Jiafeng Zhou, Yuxin Wei, Qian Cai, Yongchun Chen, Eugene Edzeafene-Mensah, Yunjun Yang, Zhifang Pan

**Affiliations:** 1 Radiology Department, The First Affiliated Hospital of Wenzhou Medical University, Nanbaixiang, Ouhai District, Wenzhou, Zhejiang, China; 2 The Eye Hospital of Wenzhou Medical University, Wenzhou, China; 3 The First Affiliated Hospital of Wenzhou Medical University, Nanbaixiang, Ouhai District, Wenzhou, Zhejiang, China, 86 15088581221; 4 School of Basic Medical Sciences, Wenzhou Medical University, Wenzhou, Zhejiang, China

**Keywords:** large language models, radiology reports, AI, error detection, quality control

## Abstract

**Background:**

Large language models (LLMs) show promise in automatically detecting errors in radiology reports, but their performance remains insufficiently validated in large-scale, real-world clinical datasets.

**Objective:**

This study aimed to systematically evaluate the performance of LLMs in detecting and correcting errors in Chinese radiology reports derived from authentic clinical data.

**Methods:**

A large-scale dataset of 4480 Chinese radiology reports with modification records containing real clinical practice-generated errors was retrospectively collected between January 2023 and June 2024 at a single institution. After exclusions, 1363 reports containing 1551 errors were included. The dataset covers various anatomical parts of the body from different imaging modalities and was randomly divided into a test set (n=1263) and an internal validation set (n=100). Additionally, 100 error-free reports were added to the internal validation set. An additional 200 English-language reports from the Medical Information Mart for Intensive Care (MIMIC-III) were used for external validation. Eight human readers and 8 widely adopted LLMs, enhanced by prompt engineering, were tasked with error detection. Overall and subgroup detection performance and reading time were evaluated. Correction suggestions from the 2 best-performing LLMs were reviewed by a senior radiologist.

**Results:**

On the test set, DeepSeek-R1 achieved the highest overall detection rate at 89% (95% CI 87%-90%), significantly better than the other 7 models (*P*=.001-.007). On the internal validation set, DeepSeek-R1 and Claude-3.5-Sonnet achieved detection rates of 83% (100/120; 95% CI 76%-89%) and 80% (96/120; 95% CI 72%-86%), respectively. DeepSeek-R1 showed performance comparable to radiologists (83%, 95% CI 76%-89% vs 80%, 95% CI 72%-86% for junior radiologists and 78%, 95% CI 70%-85% for senior radiologists; *P*=.39 and *P*=.19, respectively) and significantly better performance than that of nonradiologists and nonphysicians (83%, 95% CI 76%-89% vs 66%, 95% CI 57%-74% and 38%, 95% CI 30%-47%; *P*<.001, respectively). DeepSeek-R1 showed a false-positive rate comparable to radiologists (DeepSeek-R1 vs senior radiologists and junior radiologists, 3% vs 0% and 1%; *P*=.25 and *P*=.61, respectively) and a significantly lower rate than nonradiologists and nonphysicians (3% vs 13% and 17%; *P*=.02 and *P*=.002, respectively). On the external validation set, DeepSeek-R1 and Claude-3.5-Sonnet achieved detection rates of 94% (95% CI 89%-97%) and 93% (95% CI 88%-97%), respectively. The correction accuracy of DeepSeek-R1 and Claude-3.5-Sonnet was 95% and 91%, respectively.

**Conclusions:**

Enhanced LLMs, particularly DeepSeek-R1, demonstrated robust performance in error detection and correction within real-world Chinese radiology reports, supporting their clinical use for automated quality assurance and integration into workflows to improve reporting accuracy and efficiency.

## Introduction

Radiology reports are critical for clinical decision-making, as they facilitate the communication of complex imaging findings in a comprehensible, accurate, and efficient manner, thereby guiding patient management and treatment [1,2]. The importance of accurate radiology reports in medical practice cannot be overstated. Even minor errors in the reports have the potential to result in severe consequences, such as incorrect diagnoses or delayed treatments [3,4]. However, radiology reports are prone to errors due to unreliable speech recognition and cognitive fatigue resulting from increasing radiologist workloads and high-pressure clinical environments [3,5,6].

Large language models (LLMs), as a new AI method, can learn complex language patterns and generate fluent and coherent text, demonstrating revolutionary potential in medical processing [7-10]. GPT-4 has been demonstrated to have error detection performance comparable to board-certified radiologists and holds great promise for substantial reductions in work hours and costs [11]. This indicates that LLMs can serve as an effective quality control tool in radiology reports [12,13]. However, some critical gaps in current research hinder direct clinical application. First, most studies are based on single-language English datasets. Evidence indicates that GPT’s performance in certain tasks differs between English and non-English environments due to differences in linguistic structures, medical terminology, and contextual nuances [14,15]. Non-English radiology corpora—especially Chinese, where logographic characters, abbreviated terminologies (eg, “Ca” for cancer), and hybrid Latin-Chinese descriptors abound—pose additional lexical and semantic challenges that may degrade model performance [16]. Second, many studies used artificially constructed and simulated errors. The generated data has low transparency, is difficult to verify, and may introduce new biases such as hallucinations, overfitting, and low generalization, which will hinder the local operation of LLMs in medical institutions [2]. Third, prior work has focused almost exclusively on error detection; the capacity of LLMs to correct errors with clinically acceptable reasoning remains largely unexplored.

This study aimed to address these critical gaps by systematically evaluating the error detection and correction capabilities of prompt-enhanced LLMs using a large-scale Chinese radiology report dataset derived from real clinical workflows. We hypothesized that prompt-enhanced LLMs would have good performance in error detection and correction while maintaining high speed and acceptable false-positive rates (FPRs).

## Methods

### Ethical Considerations

This study was approved by the institutional review board of The First Affiliated Hospital of Wenzhou Medical University (approval number KY2025-R084). The requirement for written informed consent was waived because of the retrospective design. All patient identifiers were removed before the reports were provided to the LLMs and human reviewers.

### Data Collection

A total of 4480 Chinese radiology reports with modification records were obtained from the radiology system of our hospital between January 2023 and June 2024. Reports needing image re-evaluation to verify errors, diagnoses revised due to misdiagnosis, and controversial reports were excluded, resulting in a final corpus of 1363 reports containing 1551 errors (X-ray: 27, computed tomography [CT]: 1250, and magnetic resonance imaging [MRI]: 86). The dataset covers various anatomical parts of the body from different imaging modalities. The errors in the reports were all generated by radiologists during routine clinical work. The dataset was randomly divided into a test set (n=1263) and an internal validation set (n=100). Additionally, 100 error-free reports were added to the internal validation set. For further validation of the generalizability of our approach, 200 English reports from the Medical Information Mart for Intensive Care (MIMIC-III) dataset were used, including 100 reports with 148 inserted errors and 100 error-free reports (see Note S1 in Multimedia Appendix 1).

### Data Annotation

Errors in the reports were initially annotated by a junior radiologist and checked by basic LLMs (eg, DeepSeek-R1) to find potential errors (details are provided in Note S2 in Multimedia Appendix 1). The basic LLMs participated only as static annotation auxiliary tools, and their model parameters remained frozen throughout the entire process without any form of training or fine-tuning. All interactive data generated during the annotation phase were strictly limited to the inference level and did not affect model parameter updates through gradient feedback. LLMs served only as supplementary screening aids to flag potential candidate errors and did not determine the final reference standard. All annotations were subsequently reviewed by a senior radiologist who retained the authority to reject the suggestions of LLMs, thereby ensuring comprehensive error detection while minimizing the introduction of model bias. The report reviewed by the senior radiologist was the final annotated data. Errors were categorized into 5 types based on the characteristics of the Chinese language and previous studies [11]: (1) omission, (2) addition, (3) semantic error, (4) location discrepancy, and (5) others. The detailed definition of each type of error is provided in Table S1 of Multimedia Appendix 1. The severity of the errors was categorized as either “clinically significant” or “not clinically significant” in accordance with recent studies [12]. Clinically significant errors were considered to be of such magnitude that they could alter the meaning of the report, thereby risking misinterpretation by the clinician.

### Prompt Engineering

To enhance the error detection ability of LLMs, we randomly selected 100 reports from the test set for multiple iterations of model optimization. First, the LLMs were assigned the role of a professional radiologist and informed that their core task was to detect errors in radiology reports. The detection scope was then restricted using the 5 error types and enhanced by integrating chain-of-thought reasoning [17] along with a few-shot examples. In addition to thinking step by step, the model was further instructed to “split reports for sentence-by-sentence review” and to verify anatomical sites and medical terminology based on different categories. A true positive was defined as when the LLMs flagged a text segment that corresponded to errors in the reference standard (details are provided in Note S3 of Multimedia Appendix 1). To mitigate the “hallucinations” [8] phenomenon caused by LLMs’ automatic correction of diagnostic content, a clear restriction—“prohibiting modification of the original report contents”—was implemented. By using these instructions and restrictions, the accuracy of the model’s error recognition at the word level was effectively improved. The prompt templates were continuously adjusted and improved during the experimental exploration process until the model no longer showed significant improvement. The temperature was set to 0.3 based on a subset, ensuring stability and high accuracy. Our prompt template and parameters are provided in Note S4 of Multimedia Appendix 1. All of the LLMs were run once per report, with calling processes using a “single report, single conversation, no context accumulation” approach. This design ensured that the knowledge representation ability of LLMs remained unchanged, fundamentally avoiding the potential impact of data leakage on subsequent test set evaluation results.

### Study Design

**Figure 1.**
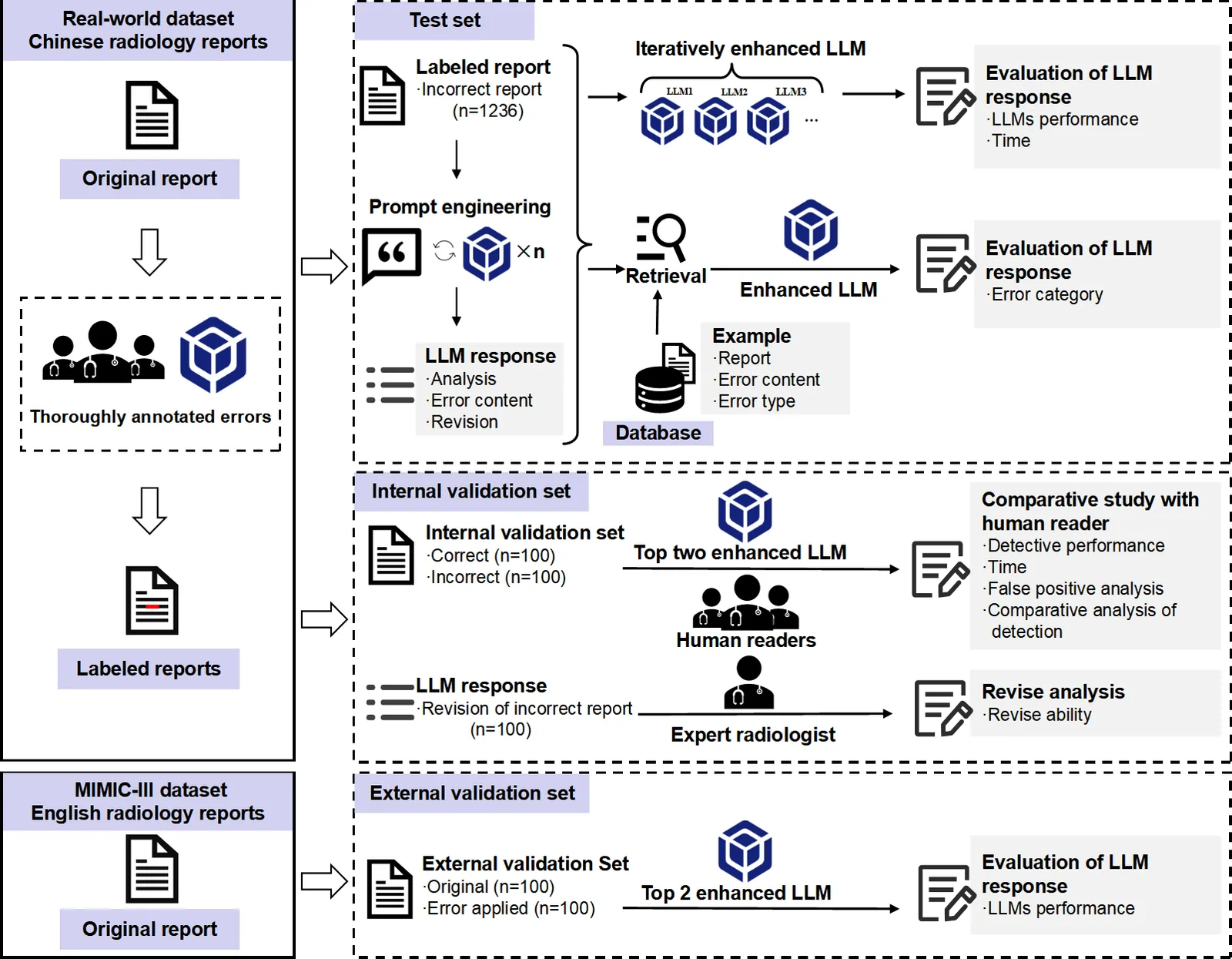
The overall structure of the study using enhanced large language model (LLMs), including annotation for Chinese radiology reports containing errors, 8 enhanced LLMs screened on the test set, comparison of the top 2 models with human readers on the internal validation set, review and validation of the correction suggestions from the LLMs by an expert radiologist, and validation of an external validation set with 200 English reports from MIMIC-III. MIMIC-III: Medical Information Mart for Intensive Care III.

To validate the application of LLMs on real clinical data, 2 experiments were conducted (Figure 1). In part 1, 8 widely used LLMs were evaluated for error detection on the test set. These included international mainstream models such as GPT-4, GPT-4o (OpenAI), Gemini-1.5-Pro (Google), Claude-3.5-Sonnet (Anthropic), and models primarily trained on Chinese corpora such as DeepSeek-V3, DeepSeek-R1 (DeepSeek), Qwen-Plus (Alibaba Cloud), and GLM-4 (Zhiyuan AI). The model’s output included the analysis, error fragment, and revision. The error detection rate was used to evaluate the performance of the models, and the time consumed was recorded from prompt submission to final response. To facilitate quality management of error detection, the LLMs’ ability to classify error types was also evaluated. A retrieval-augmented generation [18] and in-context learning [19] framework was applied, retrieving the top 3 most relevant examples per test sample to perform error-type classification based on predefined categories. This task serves as an indirect evaluation of the overall improvement in the model’s semantic understanding and reasoning abilities resulting from the proposed optimization.

In part 2, the top 2 LLMs were further analyzed on an internal validation set and an external validation set. Eight human readers from various backgrounds—including 2 senior and junior radiologists, 2 nonradiologists, and 2 nonphysicians—were compared with the LLMs in terms of error detection performance and time consumption on the internal validation set. The analysis and correction results provided by the LLMs were evaluated by an experienced radiologist. Detailed information about the human readers is provided in Note S5 of Multimedia Appendix 1.

### Statistical Analysis

Statistical analyses were performed using Python (version 3.8.19) and the pandas library (version 2.0.3, NumFOCUS). A paired test was used to assess differences between the LLMs and human readers. *P* values <.05 were considered statistically significant. Additionally, 95% CIs were calculated using the Wilson method. The model’s error detection performance was evaluated by detection rate, while its classification performance was assessed through precision, recall, and *F*_1_-score. All statistical analyses were performed at the error level, with each error treated as an independent observation rather than at the report level.

## Results

### Characteristics of the Test Set

**Figure 2.**
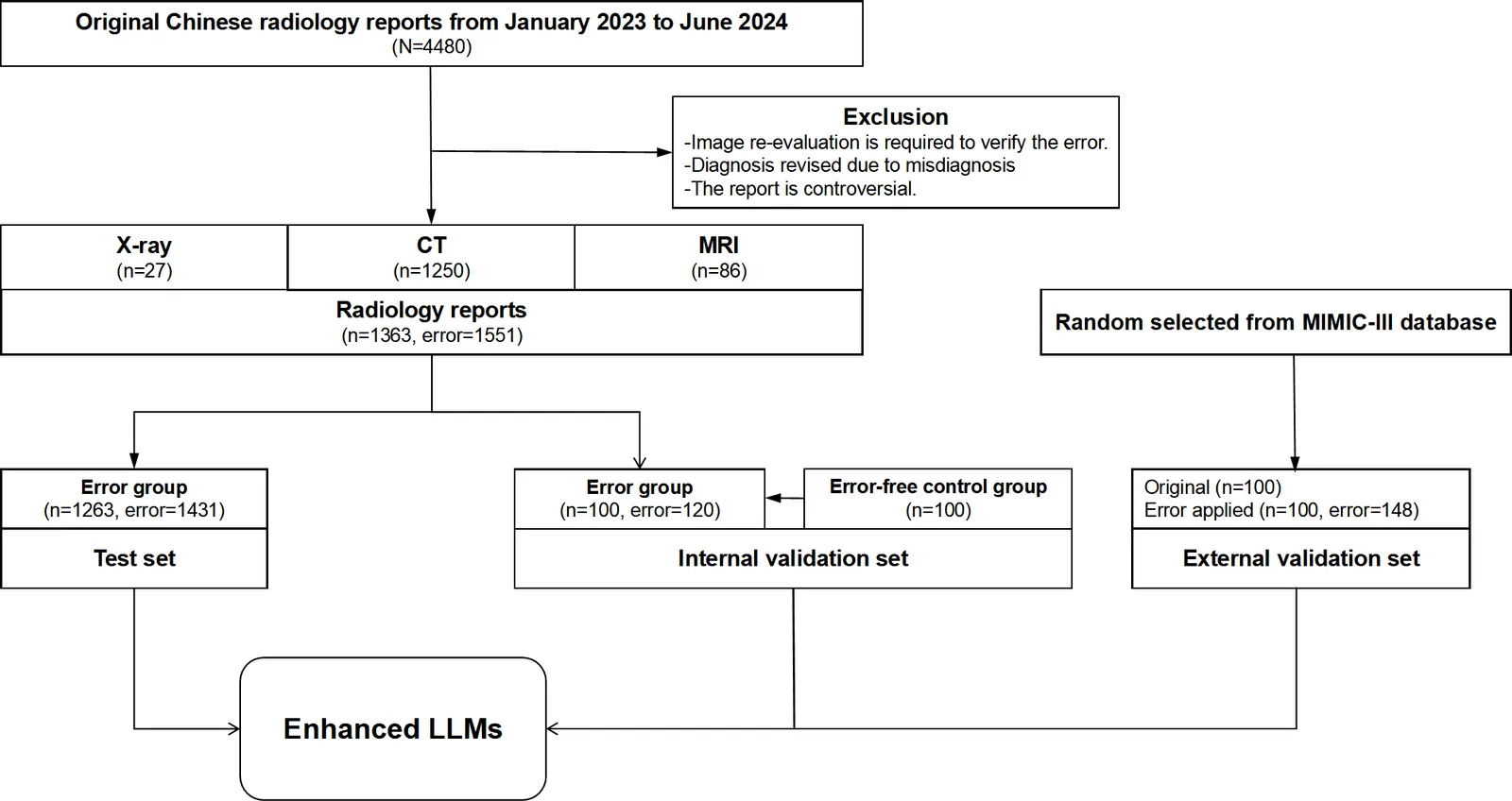
Study flowchart of real-world Chinese radiology report selection, dataset splitting, and external English validation for LLM error detection evaluation. CT: computed tomography; LLM: large language model; MIMIC-III: Medical Information Mart for Intensive Care III; MRI: magnetic resonance imaging.

The study flowchart is shown in Figure 2. The test set comprised 1263 reports containing 1431 errors, including 552 omission errors, 183 addition errors, 339 semantic errors, 202 location discrepancy errors, and 155 categorized as others. Clinically significant errors outnumbered not clinically significant errors (808 vs 623).

### Enhanced LLMs Performance

#### Error Detection

**Figure 3.**
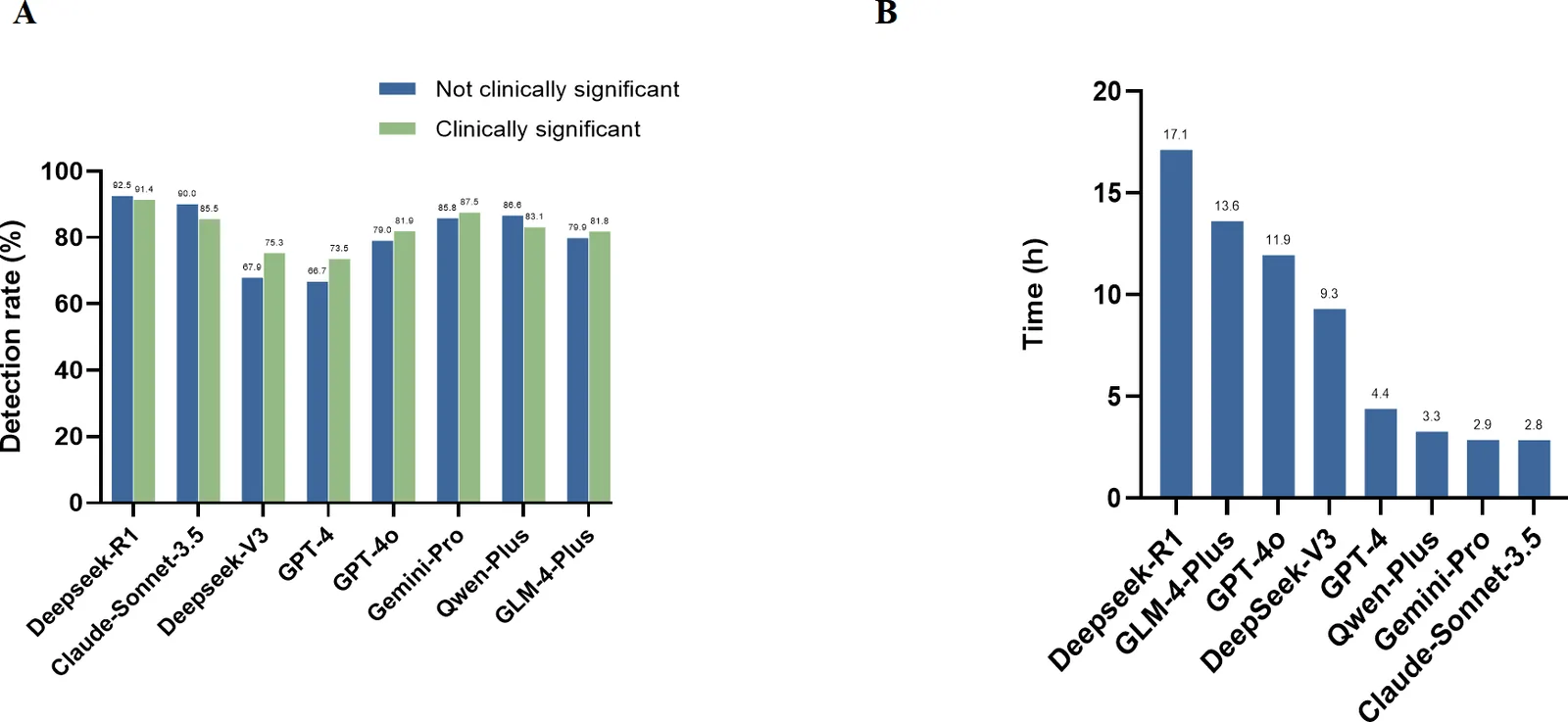
(A) The bar chart presents the detection rates of clinically significant and not clinically significant errors by large language models (LLMs). (B) The bar chart displays the total processing time by LLMs.

On the test set, the models primarily trained on the Chinese corpora had a comparable average detection rate to the international mainstream models (78%, 95% CI 77%-79% vs 77%, 95% CI 76%-78%). DeepSeek-R1 achieved the highest overall detection rate at 89% (95% CI 87%-90%), significantly better than the other 7 models (*P*=.001-.007; Table 1). Within each model, the detection rate showed no notable difference between clinically significant and not clinically significant errors (Figure 3A).

**Table 1.** The detection performance of large language models on the test set.

Model	Detection rate, % (95% CI)					
	Omission	Addition	Semantic error	Location discrepancy	Others	Total
GPT^a^-4	64 (60-68^b^)	52 (45-60)^b^	56 (51-61)^b^	85 (80-89)^b^	81 (74-87)^b^	65 (63-68)^b^
GPT-4o	80 (76-83)^b^	62 (55-69)^b^	66 (61-71)^b^	90 (85-93)^b^	84 (77-89)^b^	76 (74-78)^b^
Gemini-Pro	84 (81-87)^b^	71 (64-77)^b^	75 (70-79)^b^	96 (92-98)	87 (81-91)^b^	82 (80-84)^b^
DeepSeek-V3	72 (68-75)^b^	47 (40-54)^b^	54 (48-59)^b^	87 (82-91)^b^	74 (67-80)^b^	67 (64-69)^b^
GLM^c^4-Plus	84 (80-87)^b^	57 (50-64)^b^	65 (60-70)^b^	93 (88-95)	74 (67-80)^b^	76 (74-78)^b^
Qwen-Plus	78 (75-82)^b^	69 (62-75)^b^	79 (74-83)^b^	92 (88-95)	81 (74-86)^b^	79 (77-81)^b^
Claude-3.5-Sonnet	84 (81-87)^b^	78 (71-83)	81 (76-84)^b^	96 (92-98)	93 (88-96)	85 (83-87)^b^
DeepSeek-R1	89 (86-91)	81 (75-86)	86 (82-89)	96 (92-98)	94 (89-96)	89 (87-90)

a GPT is developed by OpenAI.

b Indicates *P*<.05.

c GLM: generative language model developed by Zhipu AI.

#### Reading Time

Claude-3.5-Sonnet consumed the shortest time (2.8 h), while DeepSeek-R1 consumed the longest time (17.1 h) on the test set (Figure 3B).

#### Error Types Classification

Overall, DeepSeek-R1, Claude-3.5-Sonnet, and Gemini-Pro were ranked among the top 3 in error classification, with *F*_1_-scores ranging from 0.87 to 0.90. DeepSeek-R1, Claude-3.5-Sonnet, and Gemini-Pro also demonstrated strong capabilities in identifying the severity of errors, with *F*_1_-scores ranging from 0.90 to 0.93 (Table S2 in Multimedia Appendix 1).

### The Generalization Ability of LLMs

On the internal validation set (Table 2), DeepSeek-R1 achieved a detection rate of 83% (100/120, 95% CI 76%-89%), and Claude-3.5-Sonnet achieved a detection rate of 80% (96/120, 95% CI 72%-86%). Except for semantic errors, where DeepSeek-R1 had significantly higher detection rates (25/27, 93% vs 20/27, 74%; *P*=.01), no statistically significant differences in detection rates were observed between DeepSeek-R1 and Claude-3.5-Sonnet in the overall and subgroup analyses (*P*=.27-.99; Tables 3-5). DeepSeek-R1 had a comparable FPR with Claude-3.5-Sonnet (3% vs 4%; *P*>.99). On the external validation set, DeepSeek-R1 achieved a detection rate of 94% (139/148, 95% CI 89%-97%), and Claude-3.5-Sonnet achieved a detection rate of 93% (137/148, 95% CI 88%-97%). DeepSeek-R1 had a lower FPR compared to Claude-3.5-Sonnet (5% vs 4%; *P*>.99).

**Table 2.** Error detection rates of the top 2 large language models on internal validation and external validation sets.

Reader	Detection rate, n/N (%)						FPR^a^
	Omission	Addition	Semantic error	Location discrepancy	Others	Total	
Internal validation set							
Claude-3.5-Sonnet	23/32 (72)	12/19 (63)	20/27 (74)	28/29 (97)	13/13 (100)	96/120 (80)	4
DeepSeek-R1	25/32 (78)	10/19 (53)	25/27 (93)	28/29 (97)	12/13 (92)	100/120 (83)	3
External validation set							
Claude-3.5-Sonnet	11/15 (73)	18/19 (95)	31/35 (89)	40/41 (98)	37/38 (97)	137/148 (93)	4
DeepSeek-R1	10/15 (67)	16/19 (84)	35/35 (100)	40/41 (98)	38/38 (100)	139/148 (94)	5

a FPR: false-positive rate.

**Table 3.** Comparison of error detection rates between large language models and human readers on the internal validation set.

Reader	Total		X-ray or MRI^a^		CT^b^	
	Detection rate, % (95% CI)	*P* value	Detection rate, % (95% CI)	*P* value	Detection rate, % (95% CI)	*P* value
Junior 1	78 (69-84)	.22	56 (34-75)	.02^c^	81 (73-88)	.71
Junior 2	82 (74-88)	.73	67 (44-84)	.27	84 (76-90)	.84
Junior radiologists average	80 (72-86)	.39	61 (39-80)	.07	83 (74-89)	.92
Senior 1	76 (67-83)	.13	78 (55-91)	.67	75 (66-83)	.13
Senior 2	80 (72-86)	.47	61 (39-80)	.04^c^	83 (75-89)	>.99
Senior radiologists average	78 (70-85)	.19	69 (44-84)	.17	79 (71-86)	.39
Nonradiologist 1	65 (56-73)	<.001^c^	50 (29-71)	.06	68 (58-76)	.007^c^
Nonradiologist 2	67 (58-74)	.002^c^	67 (44-84)	.19	67 (57-75)	.006^c^
Nonradiologists average	66 (57-74)	<.001^c^	58 (34-75)	.07	67 (57-75)	.002^c^
Nonphysician 1	46 (37-55)	<.001^c^	28 (12-51)	<.001^c^	49 (40-59)	<.001^c^
Nonphysician 2	30 (23-39)	<.001^c^	17 (6-39)	<.001^c^	32 (24-42)	<.001^c^
Nonphysicians average	38 (30-47)	<.001^c^	22 (9-45)	<.001^c^	41 (32-51)	<.001^c^
Claude-3.5-Sonnet	80 (72-86)	.42	78 (55-91)	.67	80 (72-87)	.49
DeepSeek-R1	83 (76-89)	—^d^	83 (61-94)	—	83 (75-89)	—

a MRI: magnetic resonance imaging.

b CT: computed tomography.

c Indicates *P*<.05.

d Not applicable.

**Table 4.** Comparison of detection rates for different error types in radiology reports on the internal validation set.

Reader	Omission		Addition		Semantic error		Location discrepancy		Others	
	Detection rate, % (95% CI)	*P* value	Detection rate, % (95% CI)	*P* value	Detection rate, % (95% CI)	*P* value	Detection rate, % (95% CI)	*P* value	Detection rate, % (95% CI)	*P* value
Junior 1	56 (39-72)	.08	89 (69-97)	.03^a^	78 (59-89)	.01^a^	97 (83-99)	.33	69 (42-87)	.08
Junior 2	88 (72-95)	.08	79 (57-91)	.33	89 (72-96)	.08	79 (62-90)	.33	62 (36-82)	.04^a^
Junior radiologists average	72 (55-84)	.74	84 (62-94)	.047^a^	83 (63-92)	.02^a^	88 (74-96)	.82	65 (36-82)	.047^a^
Senior 1	81 (65-91)	.45	53 (32-73)	.08	70 (52-84)	.006^a^	90 (74-96)	>.99	77 (50-92)	.17
Senior 2	66 (48-80)	.21	89 (69-97)	.03^a^	78 (59-89)	.02^a^	97 (83-99)	.33	69 (42-87)	.08
Senior radiologists average	75 (58-87)	.76	66 (41-81)	.80	74 (55-87)	.003^a^	93 (78-98)	.65	77 (50-92)	.10
Nonradiologist 1	69 (51-82)	.45	47 (27-68)	.54	56 (37-72)	<.001^a^	90 (74-96)	>.99	46 (23-71)	.008^a^
Nonradiologist 2	56 (39-72)	.14	63 (41-81)	.72	67 (48-81)	.001^a^	86 (69-95)	.71	54 (29-77)	.02^a^
Nonradiologists average	59 (42-74)	.12	58 (36-77)	>.99	59 (41-75)	<.001^a^	88 (74-96)	.81	58 (36-82)	.006^a^
Nonphysician 1	31 (18-49)	<.001^a^	32 (15-54)	.06	41 (25-59)	<.001^a^	72 (54-85)	.13	54 (29-77)	.02^a^
Nonphysician 2	6 (2-20)	<.001^a^	32 (15-54)	.03^a^	56 (37-72)	<.001^a^	24 (12-42)	<.001^a^	46 (23-71)	.008^a^
Nonphysicians average	20 (9-35)	<.001^a^	32 (15-54)	.04^a^	44 (28-63)	<.001^a^	48 (31-66)	<.001^a^	54 (29-77)	.006^a^
Claude-3.5-Sonnet	72 (55-84)	.33	63 (41-81)	.27	74 (55-87)	.01^a^	97 (83-99)	.33	100 (77-100)	.34
DeepSeek-R1	78 (61-89)	—^b^	53 (32-73)	—	93 (77-98)	—	97 (83-99)	—	92 (67-99)	—

a Indicates *P*<.05.

b Not applicable.

**Table 5.** Comparison of detection rates for the severity of errors on the internal validation set.

Reader	Not clinically significant		Clinically significant	
	Detection rate, % (95% CI)	*P* value	Detection rate, % (95% CI)	*P* value
Junior radiologist 1	77 (61-88)	.06	78 (68-85)	.84
Junior radiologist 2	86 (71-94)	.02^a^	80 (70-87)	.57
Junior radiologist average	80 (64-90)	.08	79 (70-87)	.91
Senior radiologist 1	69 (52-81)	<.001^a^	79 (69-86)	.84
Senior radiologist 2	77 (61-88)	.03^a^	81 (72-88)	.67
Senior radiologist average	71 (55-84)	<.001^a^	81 (70-87)	.55
Nonradiology physician 1	51 (36-67)	<.001^a^	71 (60-79)	.20
Nonradiology physician 2	57 (41-72)	<.001^a^	71 (60-79)	.20
Nonradiology physician average	57 (41-72)	<.001^a^	69 (59-78)	.10
Nonradiologists 1	54 (38-70)	<.001^a^	42 (32-53)	<.001^a^
Nonradiologists 2	49 (33-64)	<.001^a^	22 (15-32)	<.001^a^
Nonradiologists average	53 (36-67)	<.001^a^	32 (23-42)	<.001^a^
Claude-3.5-Sonnet	86 (71-94)	.66	78 (68-85)	.50
DeepSeek-R1	91 (78-97)	—^b^	80 (70-87)	—

a Indicates *P*<.05.

b Not applicable.

### Comparison Between LLMs and Human Readers

In the overall analysis, there were no statistically significant differences in the average performance in detection rate between DeepSeek-R1 and radiologists (DeepSeek-R1, junior radiologists, and senior radiologists: 83%, 95% CI 76%-89% vs 80%, 95% CI 72%-86% and 78%, 95% CI 70%-85%; *P*=.39 and *P*=.19, respectively). DeepSeek-R1 had a significantly higher average detection rate than nonradiologists and nonphysicians (83%, 95% CI 76%-89% vs 66%, 95% CI 57%-74% and 38%, 95% CI 30%-47%; *P*<.001, respectively; Table 3).

In the subgroup analysis of different imaging modalities, there were no statistically significant differences in the detection rates of X-ray or MRI and CT reports between DeepSeek-R1 and radiologists (*P*=.07-.99). The detection rate of DeepSeek-R1 was significantly higher than the average detection rate of nonradiologists average in CT reports (83%, 95% CI 75%‐89% vs 67%, 95% CI 57%‐75%; *P*=.002) and nonphysicians average in CT and X-ray or MRI reports (83%, 95% CI 75%‐89% vs 22%, 95% CI 9%‐45% and 41%, 95% CI 32%‐51%; *P*<.001 and *P*<.001, respectively; Table 3).

The performance of DeepSeek-R1 in detecting addition errors was significantly worse than that of the best-performing radiologist (53%, 95% CI 32%-73% vs 89%, 95% CI 69%-97%; *P*=.03; Table 4). The performance of DeepSeek-R1 was significantly higher than that of nonradiologists in detecting semantic errors (93%, 95% CI 77%-98% vs 59%, 95% CI 41%-75%; *P*<.001) and significantly higher than that of nonphysicians in detecting omission, semantic, and location discrepancy errors (78%, 95% CI 61%-89% vs 20%, 95% CI 9%-35%; *P*=.01; 93%, 95% CI 77%-98% vs 49%, 95% CI 28%-63%; *P*<.001; 97%, 95% CI 83%-99% vs 48%, 95% CI 31%-66%; *P*<.001; Table 4). There were no statistically significant differences in detection rates in the analysis of error severity between DeepSeek-R1 and radiologists (*P*=.11-.99). The performance of DeepSeek-R1 was significantly higher than that of nonradiologists and nonphysicians in detecting not clinically significant errors (91%, 95% CI 78%-97% vs 57%, 95% CI 41%-72% and 53%, 95% CI 36%-67%; *P*<.001 and *P*<.001, respectively), and significantly higher than nonphysicians in detecting clinically significant errors (80%, 95% CI 70%-87% vs 32%, 95% CI 23%-42%; *P*<.001; Table 5).

**Figure 4.**
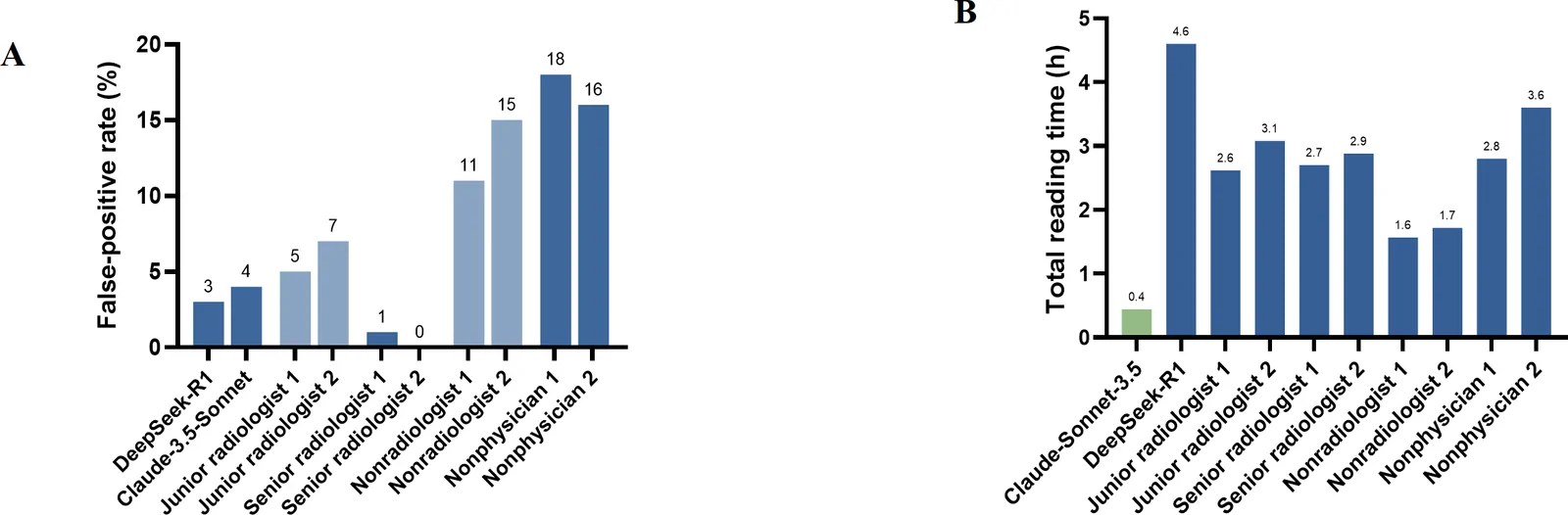
(A) False-positive rate of different human readers and large language models (LLMs). (B) Total reading time of LLMs and human readers.

The FPR did not show statistically significant differences between DeepSeek-R1 and radiologists (DeepSeek-R1 vs senior radiologists and junior radiologists: 3% vs 0% and 1%; *P*=.25 and *P*=.61, respectively). DeepSeek-R1 demonstrated a significantly lower FPR than nonradiologists and nonphysicians (3% vs 13% and 17%; *P*=.02 and *P*=.002, respectively; Figure 4A).

The total reading time of DeepSeek-R1 was 4.62 hours, significantly longer than that of the slowest nonphysician (4.62 h vs 3.6 h; *P*=.01). The total reading time of Claude-3.5-Sonnet was 0.44 hours, significantly shorter than that of the fastest nonradiologists (0.44 h vs 1.56 h; *P*<.001; Figure 4B).

### Error Reasoning and Revision Quality

**Figure 5.**
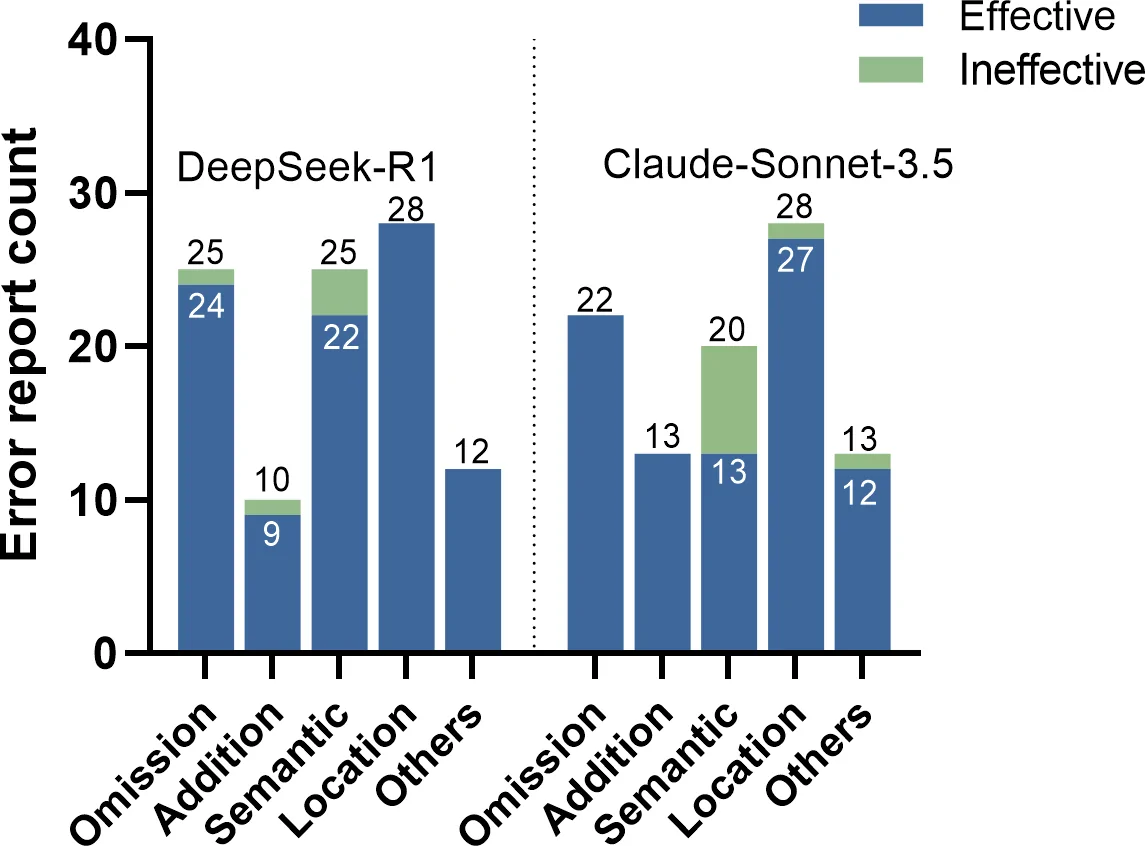
Evaluation of the validity of modification suggestions provided by the model for detected errors in reports.

The correction accuracy of DeepSeek-R1 and Claude-3.5-Sonnet was 95% and 91%, respectively (Figure 5). The correction accuracy of DeepSeek-R1 in location discrepancy and other errors, as well as the correction accuracy of Claude-3.5-Sonnet in omission and addition errors, were all 100%.

## Discussion

### Principal Findings

This study systematically evaluated the ability of mainstream LLMs to detect and correct errors in Chinese radiology reports using a large-scale, real clinical scenario dataset. Enhanced by domain-specific prompts, chain-of-thought reasoning, and strict output constraints, DeepSeek-R1 achieved detection rates of 89% (95% CI 87%-90%), 83% (95% CI 76%-89%), and 94% (95% CI 89%-97%) across test, internal, and external validation sets, respectively, matching radiologists and outperforming nonradiologists and nonphysicians. Moreover, DeepSeek-R1 and Claude-3.5-Sonnet also corrected 95% and 91% of flagged errors, respectively. These findings offer new perspectives on the localization and application of LLMs for automated quality control in digital health care settings.

### Comparison with Prior Work

Our study expanded the application of LLMs to non-English environments by using the largest error-labeled Chinese radiology report dataset (1363 reports, 1551 real errors). Our prompt engineering strategy, integrating chain-of-thought reasoning and strict output constraints, was crucial in mitigating hallucinations and focusing models on detection, thereby enhancing their reliability for clinical integration [9,17]. We found that DeepSeek-R1 achieved an 89% (95% CI 87%-90%) detection rate on the test set and 83% (95% CI 76%-89%) on the internal validation, translating to a 25% to 30% absolute gain over the 52% zero-shot ceiling reported by Yan et al [20] for Claude-3.5-Sonnet in Chinese ultrasound reports. Moreover, DeepSeek-R1 achieved a 94% (95% CI 89%-97%) detection rate on the English radiology reports dataset (MIMIC-III) without retraining, suggesting that the core optimization framework is transferable across languages, mitigating the single-language limitation. On the contrary, international mainstream models such as the GPT-4 series exhibited only moderate performance. This disparity underscores the critical impact of linguistic and domain-specific adaptation. Chinese radiology reports present features including word segmentation ambiguity, prevalent terminology abbreviations (eg, “Ca” for cancer), variable omission of quantifiers, and context-dependent semantic expressions. These unique challenges demand a deeply localized LLM solution. DeepSeek-R1, pretrained on high-quality Chinese corpora rich in medical literature, explicitly embeds domain-specific knowledge through adaptive training [21]. This design stands in contrast to GPT-4, whose training data is predominantly English and lacks publicly disclosed optimization for Chinese medical text [22]. The present study highlights that integrating domain-specific knowledge and prompt engineering can unlock robust error detection for non-English clinical narratives while remaining transferable to English data.

A significant advancement of this study was the use of a real-world dataset, as opposed to artificially constructed or synthetic data prevalent in prior research. Gertz et al [11] intentionally inserted 150 errors from 5 common error categories into 100 reports and found that the performance of GPT-4 was comparable to that of radiologists. Sun et al [4] highlighted the potential of fine-tuned LLMs to enhance error detection using LLM-generated synthetic datasets. Although such reports offer better control and avoid privacy concerns and the bias present in real-world data, they may generate new biases, such as overfitting and low generalizability [2]. There may be poor performance in real life if the generated data does not represent a few common scenarios. Other limitations include the low transparency of generated datasets, the risk of perpetuating human-generated bias, and the difficulty of validating them [23]. It should be pointed out that we used artificially inserted errors as the external validation set, which may also not fully represent the complexity and subtlety of real-world English radiology report errors. To bridge these gaps, our primary Chinese dataset captures 1551 naturally occurring errors extracted from the routine radiology workflow, ensuring that the evaluated performance is representative of genuine clinical scenarios, enhancing the practical applicability of our findings.

Compared with human experts, enhanced LLMs, particularly DeepSeek-R1, demonstrated strong error-detection capabilities in radiology reports, surpassing the performance of human readers and matching radiologists on certain tasks. In addition, DeepSeek-R1 performed well in error-type classification and maintained stable detection across imaging modalities with a low FPR. These findings align with prior studies [20] and support their potential as assistive tools for quality assurance, especially amid growing clinical workloads. To better apply LLMs to clinical practice, in addition to considering detection rate, time consumption should also be taken into account. We found that DeepSeek-R1 achieved a 4% increase in detection rate compared to Claude-3.5-Sonnet but required 6 times the processing time. DeepSeek-R1 has 671 billion parameters, 61 layers of transformers embedded with multihead latent attention and mixture-of-experts layers, generating thousands to tens of thousands of internal reasoning tokens before producing the final answer, resulting in significantly longer output times [21]. However, Claude-3.5-Sonnet prioritizes speed through architectural simplicity and implicit inference. On the contrary, when this study was conducted, DeepSeek-R1 was priced at US $0.55 per million input tokens, which was significantly lower than Claude-3.5-Sonnet, priced at US $3.00 per million input tokens. Thus, low-cost DeepSeek-R1 may be more suitable for real-world implementation decisions, such as offline quality control rather than real-time assistance. In addition, previous studies have shown that LLMs achieved a lower mean correction cost per report than the most cost-efficient radiologist [11]. Future efforts should focus on optimizing the balance between accuracy and efficiency through model compression and fine-tuning.

Notably, DeepSeek-R1 offers transparency and can be deployed locally within institutional information technology environments at substantially lower costs than proprietary models [21,24]. For local LLM deployment, there are still many challenges that need to be addressed. The first challenge is hardware constraints (eg, graphical processing unit [GPU] memory, input/output [I/O] bottlenecks) and integration hurdles (eg, heterogeneous picture archiving and communication system [PACS] interfaces). We can address these through elastic compute scaling, model quantization, domestic hardware alternatives, tiered storage, containerized deployment, and standardized HL7/FHIR (Health Level 7 International/Fast Health Care Interoperability Resources) application programming interfaces (APIs). For data security, a “data never leaves the hospital” architecture can be enforced using on-premises GPU inference, SM4/AES-256 encryption, automatic deidentification, retrieval-augmented generation for continuous knowledge updates, and confidence-based filtering to mitigate hallucinations. On the personnel and organizational front, nonintrusive user interfaces (inline highlighting, sidebar summaries) and explainable outputs (eg, error-type justifications) foster radiologist trust. The LLM only suggests corrections and deliberately disables auto-correction; the radiologist retains full authority to accept, modify, or reject each suggestion. Figure S1 in Multimedia Appendix 1 illustrates the proposed clinical workflow: (1) radiologist completes report; (2) LLM runs in background (local deployment); (3) if no error detected → report finalized; (4) if error detected → LLM highlights error and suggests correction; (5) radiologist reviews and then accepts, rejects, or modifies; and (6) final report signed. By integrating via a local API, it eliminates the need to transfer protected data or retrain models, ensuring both high performance and privacy compliance, which can be integrated into radiology reporting systems and effectively handle real-world medical data in clinical practice (Figure S2 in Multimedia Appendix 1).

A critical consideration for the clinical deployment of any AI-assisted error detection tool is its FPR, as excessive false alarms can undermine trust, increase radiologist workload, and lead to “alert fatigue” [20]. We found that DeepSeek-R1 achieved an FPR of 3% on internal validation, which was comparable to radiologists and significantly lower than nonradiologists and nonphysicians. This indicates that enhanced LLMs can maintain high specificity and minimize unnecessary interruptions for clinicians. However, certain errors, such as minor diagnostic omissions and retained normal templates, were still missed (see Table S3 in Multimedia Appendix 1). This necessitates that the final adjudication remain with the radiologist, reinforcing a human-in-the-loop paradigm where the AI acts as a sensitive assistant, not an autonomous arbiter.

Explainability is critical in health care AI to foster trust and support clinical decision-making. Prior studies have largely focused on error detection without evaluating LLMs’ ability to correct errors, which limits their explainability [11,12]. Kim et al [25] demonstrated that GPT-4 could effectively revise head CT reports. Similarly, we found that DeepSeek-R1 achieved 95% accuracy in proposed corrections. Our LLMs can provide an analysis process, reasons for correction, and suggestions for correction to increase clinical applicability. Radiologists can understand the entire correction process in order to make better decisions (accept, modify, or reject). We conducted a comprehensive qualitative analysis of all corrections and found that the LLMs were reasonable for most types of corrections, including simple typographic or grammatical, intermediate terminology or lateralization, and complex semantic rephrasing. However, there are still a small number of corrections that cannot be accepted, such as the use of professional terminology, general conclusions, specific writing habits, and so on. Representative examples of successful corrections and failed corrections were added in Table S3 in Multimedia Appendix 1. The accuracy of correction can be continuously improved by providing real-time feedback to the LLMs on the modifications and rejections provided by radiologists, in order to achieve personalized service levels. These results indicate that LLMs can support an interactive learning environment for residents by identifying common errors and providing real-time feedback, thereby promoting continuous education [11].

### Limitations

This study has several limitations. First, its retrospective design may introduce selection bias. Second, error annotation was restricted to the text level; detecting imaging-report discordance still requires visual input. Future integration of vision-language models could enable end-to-end quality assurance. Third, LLM hallucination remains a key concern, undermining clinical reliability and necessitating expert verification. Although illusions were mitigated to some extent via lower temperature and prompt constraints, fundamental solutions will require greater improvements in model architecture, knowledge injection, and training data control. Fourth, it should be noted that the external validation set (MIMIC-III) used artificially inserted errors, whereas our primary Chinese dataset contained real clinical errors. This difference may affect the generalizability of the cross-language comparison. Although the high detection rates on MIMIC-III (94% for DeepSeek-R1) suggest that the prompt engineering framework is transferable across languages, the artificial nature of these errors may not fully represent the complexity and subtlety of real-world English radiology report errors. Therefore, our cross-language findings could be interpreted as preliminary evidence of generalizability. Future work should validate on real English clinical errors to confirm these observations. Finally, prompt optimization enhances performance but poses standardization challenges and may yield variable effects across models.

### Conclusions

Based on a large-scale and real-world clinical corpus, this study demonstrates that enhanced LLMs, particularly DeepSeek-R1, can effectively detect and correct errors in Chinese radiology reports. The findings validate the use of multilingual, clinically derived data and support the local deployment of open-source LLMs as transparent, cost-effective, and privacy-preserving tools within radiology workflows, thereby facilitating their transition toward clinical decision support. While our findings support the potential clinical use of LLMs for error detection, prospective multicenter validation and workflow-based safety assessment are required before routine implementation. In the future, we will conduct multicenter validation to assess generalizability and explore multimodal LLMs for verifying image-text consistency, advancing toward AI-driven quality control in practice.

## Supplementary material

10.2196/94689 Multimedia Appendix 1 Supporting notes, tables, and figures providing additional methodological details, evaluation results, and workflow illustrations.

## Abbreviations

API: application programming interface
CT: computed tomography
FPR: false-positive rate
GPU: graphical processing unit
HL7/FHIR: Health Level 7 International/Fast Health Care Interoperability Resources
LLM: large language model
MIMIC-III: Medical Information Mart for Intensive Care III
MRI: magnetic resonance imaging
PACS: picture archiving and communication system
