# Supplementary material for: Error Detection and Correction in Chinese Radiology Reports Using Large Language Models: Real-World Clinical Validation Study
Source: J Med Internet Res. 2026 Aug 21;28:e94689. doi: 10.2196/94689 (PMC13495996; doi:10.2196/94689)
Supplement: Supplementary file 1 [file jmir-v28-e94689-s001.docx]

**Note S1**

**External English Validation Set**

The external validation set was obtained from the Medical Information Mart for Intensive Care III (MIMIC-III), a publicly available critical-care database comprising reports from 53,150 patients admitted to the intensive care units of Beth Israel Deaconess Medical Center^1^, which is among the most comprehensive resources for intensive-care research. Radiology reports were randomly sampled from MIMIC-III and screened to curate a clean analytic set.

We excluded procedure-related reports; any record flagged as erroneous by clinicians via the database field “ISERROR”^2^; reports missing either the “Findings” or “Impression” sections; reports that combined results from multiple imaging examinations within a single document; and reports that explicitly incorporated prior comparisons (e.g., statements such as “consistent with the prior examination”).

To ensure accuracy and measurability, all candidate reports that passed database-level screening underwent manual error checking by experienced physicians, and only those verified to be error-free were retained, yielding a corpus of 200 radiology reports. For validation, we then randomly selected 100 of these curated reports and injected 1–3 errors per report, with each error applied from the five predefined categories described earlier in the manuscript. These manipulated reports, together with the remaining error-free set, were used to evaluate the method’s practical utility in real-world clinical reporting across different language settings.

**Note S2**

**Data Annotation**

The errors and their types in the radiology reports were first annotated by a junior radiologist. Before annotating, the junior radiologist underwent training to ensure familiarity with the various error types commonly encountered in radiology reports. A random sample of 100 radiology reports was independently annotated by both the junior and senior radiologists, and inter-rater agreement was assessed using Cohen's Kappa (K), yielding a value of 0.726 (95% CI: 0.5646–0.8678). Then, the annotated reports were checked by a basic LLM (e.g., DeepSeek-R1) to find potential errors. The basic LLM was employed solely as an error candidate tool and utilized a "single report, single conversation, no context accumulation" approach to avoid the potential impact of data leakage that could compromise subsequent test set evaluation. LLMs did not retrieve any other report or label information in each round of calls. LLMs only participated as static annotation auxiliary tools, and their model parameters remained frozen throughout the entire process without any form of training or fine-tuning. All interactive data generated during the annotation phase were strictly limited to the inference level and didi not affect model parameter updates through gradient feedback. This design ensured that the knowledge representation ability of LLMs remained unchanged during the annotation process, thereby fundamentally preventing potential data leakage from influencing subsequent test set evaluation results. In addition, LLMs served only as a supplementary screening aid to flag potential candidate errors and did not determine the final reference standard. All annotations were subsequently reviewed by a senior radiologist who retained the authority to reject the LLMs' suggestions, thereby ensuring comprehensive error detection while minimizing the introduction of model bias. The senior radiologists were required to review reports in the following sequence: the original report, the junior radiologist annotated reports, and the basic LLMs modified reports. These measures collectively minimized the potential introduction of model bias.

**Note S3**

**True positive**

A true positive was defined as when the LLMs flagged a text segment that corresponded to errors in the reference standard. When the LLMs flagged a text segment that did not correspond to any error in the reference standard than considered a false positive. If the LLMs correctly identified an error but misclassified its error type (e.g., flagged a semantic error as an omission), it was still counted as a true positive, because error-type classification served only an auxiliary quality-management purpose; the primary objective of this study was error detection, not subtyping.

Handling of partially correct detections: When the LLMs identified the correct error but at an imprecise text span (e.g., flagging a few words adjacent to the ground-truth error), we cross-referenced the LLMs’ textual explanation/analysis output with the reference standard. If the explanation demonstrated that the LLMs had genuinely understood and identified the error (as opposed to coincidentally flagging adjacent text), the detection was counted as a true positive. If the explanation indicated that the LLMs had identified a different issue or provided no coherent rationale, it was counted as a false positive.

**Note S4**

**Prompt Template**

You are a radiology expert. Based on the given criteria, you can identify errors in radiology reports, which include [Findings] and [Impression]. Please strictly check the report for errors according to the following classification system and [Precautions].

{Five error type scenario}

[Precautions]:

1. Conduct a detailed analysis of the input text, ensuring no modification, addition, or deletion of any text, including non-professional expressions.

2. The report may involve multiple sites or multiple lesions.

3. Optimized expressions and improvements are not considered [Error Text].

4. Conclusions should concisely summarize abnormalities and do not require diagnostic details.

Next, you will be provided with a radiology report.

Let's think step by step.

Please output in the following JSON format:

[{

“Analysis”: “Divide the text into sections based on ‘。’, and analyze each section one by one.”

“Error text”: “Clearly point out the identified errors or state 'No errors'”

“Revision Suggestion”: “Provide concise suggestions for correcting the errors.”

}]

**Detailed scenarios of Error Types**

Due to the complexity of Chinese clinical contexts and the simplification of report content by physicians handling high patient volumes, generic prompts often lead to misinterpretation. Although such content may be deemed non-standard by models, it is widely accepted by physicians. Therefore, we have established specific criteria for error types to address these scenarios.

Category 1: Key abnormalities mentioned only in the Findings section but not reflected in the Conclusions. Not considered errors if: the abnormality is expressed in any form in the Conclusions; the key abnormality can be reasonably explained by the diagnosis/recommendation in the Conclusions; it is a secondary change related to the diagnosis; the abnormality has no independent significance for current clinical decision-making; the abnormality described in the Findings is a typical/direct sign of a lesion already diagnosed in the Conclusions (e.g., increased density in the sulci suggesting subarachnoid hemorrhage).

Category 2: Any arbitrary character combinations unrelated to medical descriptions; key abnormalities mentioned only in the Conclusions. Image localization markers (e.g., "img99") and diagnoses based on the described imaging findings are not considered errors.

Category 3: Major professional expression errors (e.g., "alveolar wall thickening" mistaken as "alveolar membrane thickening"); typos affecting understanding (e.g., "病炤" should be "病灶"); clearly repetitive/incomplete expressions; grammatical errors. Not considered errors if: descriptive terms used interchangeably with standard diagnostic terms (e.g., "spot-like dense shadow" and "calcification"); synonyms or near-synonyms (e.g., "cystic/cyst-like/cystoid"); empirical inferences based on imaging findings (e.g., diagnosing "streaky high-density shadow" as "inflammatory focus"); diverse expressions of professional terms (e.g., using "ca" for "cancer").

Category 4: Contradictory statements (e.g., "no abnormalities but lesions exist"); substantive contradictions between Findings and Conclusions on key conclusions; location discrepancies; recommended examination site inconsistent with described abnormalities (e.g., describing "liver" but recommending "chest enhanced" examination). Not considered errors: location differences between Findings and Conclusions pertain to anatomical subregions of the same pathology (e.g., "middle lobe and upper lobe of right lung and upper lobe of left lung" and "middle lobe of right lung and upper lobes of both lungs").

Category 5: Other errors, including but not limited to: using incorrect units in the report (e.g., writing "m" instead of "mm"); unreasonable size ranges; incomplete sizes (e.g., "diameter about mm"); date errors.

**Note S5**

**Human Information**

The errors and their types in the radiology reports were first annotated by a junior radiologist (CC, with 6 years of experience).

The annotated reports were reviewed again by a senior physician (CYC, 17 years of experience).

Eight human readers from various backgrounds—including two senior (ZJF and ZKK, 13 years and 15 years), two junior radiologists (NJN and ZMJ, 2 years and 5 years), two non-radiologists (YYH and BZL, 10 years and 3 years), and two non-physicians (XSY and WYX). The analysis and correction results provided by the LLMs were evaluated by an experienced radiologist (CYC, 17 years).

**Table S1:Types of Errors.**

| **Category** | **Definition** | **Example** | **Explanation** |
| --- | --- | --- | --- |
| Omission | Refers to the omission of relevant expressions that are only mentioned in the findings. | Findings：A few cord-like shadows are seen in the lower lobe of the left lung, and ***dense shadows are observed in the coronary arteries*.**  Impression：A few inflammatory foci in the lower lobe of the left lung. | The diagnosis of coronary artery dense shadow was omitted. |
| Addition | Insertion of expressions irrelevant encompasses redundant descriptions in impression and contradictory statements | Findings：Multiple cystic lesions are observed in the liver.  Impression：Presence of hepatic cyst and ***liver cirrhosis*.** | The impression includes liver cirrhosis, which was not found in the findings. |
| Semantic Error | Pertains the misapplication of professional terminology, including spelling mistakes, terminology error, incomplete expressions, and errors that can hinder clarity | Impression：Right kidney**“解释”** | “结石” misspelt as “解释” |
| Location Discrepancy | Refers to substantial contradictions between findings and impression, incorrect terms are describe the same anatomical body region | Findings：The ***left kidney*** shows a cystic low-density shadow.  Impression：  ***Right kidney*** cyst. | Findings described the left kidney lesion, but the impression is that the right kidney. |
| Others | Errors that do not fall under the aforementioned categories, including those related to measurement results, date errors, punctuation mistakes, and so on | A solid nodule can be seen in the upper lobe of the left lung, about 7x8***mmm*** in size. | The size should be 7x8mm. |

Note: Items in italics indicate the presence of specific error types.

**Table S2.Detailed Metrics of Error Types Classification**

| **Metric** | **Claude-3.5-sonnet** | | | **Gemini-pro** | | | **Deepseek-R1** | | |
| --- | --- | --- | --- | --- | --- | --- | --- | --- | --- |
|  | Precision | F1 | Recall | Precision | F1 | Recall | Precision | F1 | Recall |
| Total | 0.91 | 0.90 | 0.90 | 0.91 | 0.90 | 0.90 | 0.88 | 0.87 | 0.88 |
| Omission | 0.97 | 0.96 | 0.97 | 0.96 | 0.98 | 0.97 | 0.94 | 0.97 | 0.95 |
| Addition | 0.44 | 0.47 | 0.46 | 0.53 | 0.53 | 0.53 | 0.42 | 0.29 | 0.34 |
| Semantic Error | 0.89 | 0.87 | 0.88 | 0.95 | 0.89 | 0.84 | 0.93 | 0.83 | 0.88 |
| Location Discrepancy | 0.89 | 0.94 | 0.92 | 0.82 | 0.88 | 0.94 | 0.78 | 0.85 | 0.81 |
| Others | 0.97 | 0.94 | 0.95 | 0.94 | 0.96 | 0.97 | 0.94 | 1.00 | 0.97 |
| Not Clinically Significant | 0.94 | 0.93 | 0.93 | 0.91 | 0.89 | 0.88 | 0.88 | 0.85 | 0.84 |
| Clinically Significant | 0.93 | 0.93 | 0.93 | 0.92 | 0.92 | 0.92 | 0.91 | 0.90 | 0.90 |

**Table S3.Example of False Negative Result**

| Findings: No clear abnormal density was found in the bilateral cerebral hemispheres, cerebellar parenchyma, brainstem, and diencephalon. The gray-white matter contrast in the cerebral hemispheres was normal, and the morphology and size of the ventricular system were normal. The size and morphology of the sulci, fissures, and ventricles were normal, and there was no displacement of the midline structure. **Also, the nasal septum is slightly deviated to the left.**  A ground glass nodule with a diameter of about 3-4mm is seen in the lower lobe of the right lung (thin layer img96), and a few cord shadows are seen in the middle lobe of the right lung. The trachea and bronchi are unobstructed; No obvious enlarged lymph node shadows were found in the hilum and mediastinum of both lungs; The size and shape of the heart remain normal, with no abnormalities in the pericardium; No pleural effusion was found on both sides.  Impression: No significant abnormalities were found on the cranial CT plain scan. Nodule in the lower lobe of the right lung, possibly inflammatory, recommended for annual CT follow-up. There is a small fibrous lesion in the middle and lower lobes of the right lung. | **Unrecognized**  The Findings described that the nasal septum is slightly deviated to the left, but it did not appear in the Impression. |
| --- | --- |
| Findings: Localized increase in translucency was observed beneath the pleura at the apex of both lungs; No obvious abnormal density lesions were found in the remaining lung field, and the trachea and bronchi were shown to be unobstructed; No obvious enlarged lymph node shadows were found on the two lung hilum and mediastinum plain scans; The size and shape of the heart remain normal, with no abnormalities in the pericardium; No pleural effusion was observed on both sides.  Several cystic low-density shadows were observed in the liver, with the largest one measuring approximately 16mm in diameter and clearly defined. The size and morphology of the **liver**, spleen, pancreas, kidneys, and adrenal glands are normal, and no obvious abnormal density shadows are observed on plain scan. No obvious wall thickening, lumps, or stones were found in the gallbladder and bile duct, and no signs of abnormal dilation were observed. No lumps or enlarged lymph nodes were found in the abdominal cavity or retroperitoneal area within the scanning range. No fluid accumulation was found in the abdominal cavity.  Impression: Pulmonary emphysema at the apex of both lungs. Multiple cysts in the liver. | **Unrecognized**  The Findings has described the presence of cysts in the liver, but the template for the normal liver has not been removed in the following text |

**Table S4.Example of Successful and Failed Corrections**

| **Radiology report** | **Analysis** | **correction suggestions** | **Radiologist's opinion** | |
| --- | --- | --- | --- | --- |
| 发现：双侧大脑半球、小脑实质、脑干及间脑未见明确异常密度，大脑半球灰白质对比正常，脑室系统形态及大小正常，脑沟、脑裂及脑池大小及形态正常，中线结构无移位。 两肺散在实性小结节，径约2-5mmm（大者im 69），右肺中叶见少许条索影；气管、支气管显示通畅；两肺门、纵隔平扫未见明显肿大淋巴结影；心脏大小、形态如常，心包膜无殊；两侧胸腔未见积液影。右肝甲状腺见结节状低密度影。右乳腺见致密影。 右肝见钙化灶，脾脏、胰腺、肾脏、肾上腺大小形态如常，平扫未见明显异常密度影。胆囊、胆管未见明显壁增厚、肿块或结石，亦未见异常扩张等征象。所见胃肠道充盈可，未见明确壁增厚或肿块影。扫描范围内腹腔、腹膜后区未见肿块或肿大淋巴结。腹腔未见积液。 结论：颅脑CT平扫未见明显异常。 两肺散在小结节，炎性可能，建议年度CT复查。 右肺中叶少许纤维灶；右甲状腺结节、右乳腺钙化灶，请结合超声。 右肝钙化灶。 | 1. "两肺散在实性小结节，径约2-5mmm（大者im 69）" - There is a unit writing error of "mmm"   2. "右肝甲状腺见结节状低密度影" - "右肝甲状腺" is an incorrect expression and should be described separately   3. "右乳腺见致密影...右乳腺钙化灶" - It is inconsistent with the conclusion description, one area is called "致密影", and the conclusion is called "钙化灶" | 1. "径约2-5mm" 2. "右甲状腺"和"右肝"Should be described separately 3. Unified use of the description of"右乳腺钙化灶" | 1.Accept 2.modify："右肝甲状腺"modified to"右侧甲状腺" 3.Refuse, no need to modify |  |
| 发现：双侧大脑半球、小脑实质、脑干及间脑未见明确异常密度，大脑半球灰白质对比正常，脑室系统形态及大小正常，脑沟、脑裂及脑池大小及形态正常，中线结构无移位。 右肺中叶少许条索影、斑点影；余肺透亮度良好，内未见明显异常密度灶，气管、支气管显示通畅；两肺门、纵隔未见明显肿大淋巴结影；心脏大小、形态如常，心包膜无殊；两侧胸腔未见积液。右乳见斑点致密影。 两肾肾窦见少许结节状致密影；子宫未见显示；肝脏、脾脏、胰腺、肾上腺大小形态如常，平扫未见明显异常密度影。胆囊、胆管未见明显壁增厚、肿块或结石，亦未见异常扩张等征象。膀胱充盈欠佳。腹盆腔、腹膜后区未见肿块或肿大淋巴结。腹盆腔未见积液。 结论：颅脑CT平扫未见明显异常。 右肺中叶少许炎性纤维灶。 左乳微小钙化灶，请结合钼靶及超。 两肾结石。 子宫未见显示，请结合临床。 | 1. It is found that there is a description of "右乳见斑点致密影",but the conclusion mentions "左乳微小钙化灶",indicating a contradiction in the left and right positions. | It is necessary to unify the left and right positions of breast lesions, and it is recommended to verify the original image to determine the accurate location. | Accept：Unified modified to left breast |  |
| 发现：两额顶叶、侧脑室旁及基底节区见少许模糊斑片样低密度灶，余脑实质未见明确异常密度，大脑半球灰白质对比正常，脑室系统形态及大小正常，脑沟、脑裂及脑池大小及形态正常，中线结构无移位。 两肺见散在微小结节，部分呈磨玻璃密度，界清，径约2-3mm；左肺舌段见少许模糊斑片影；气管、支气管显示通畅；两肺门、纵隔未见明显肿大淋巴结影；心脏大小、形态如常，心包膜无殊；两侧胸腔未见积液。 另示：肝及右肾上腺内见斑点状致密影。左肾见脂性小结节。 结论：脑内散在曲线灶。 两肺散在微小结节，炎性考虑，随访。 左肺舌段少许炎性灶。 肝及右肾微小钙化灶；左肾小错构瘤。 | 1. "两额顶叶、侧脑室旁及基底节区见少许模糊斑片样低密度灶"-The conclusion is summarized as "脑内散在曲线灶", which is a professional error. "曲线灶" is not a standard description. 2. "肝及右肾上腺内见斑点状致密影"-The conclusion states"肝及右肾微小钙化灶"，but there is inconsistency in the location description，The findings mention "肾上腺" but the conclusion changes it to"肾"。 | 1. "脑内散在曲线灶"modified to"脑内散在低密度灶" 2. "肝及右肾微小钙化灶"modified to"肝及右肾上腺微小钙化灶" | 1.Accept 2.Accept |  |
| 发现：肝门区-胰颈上方见团块影，大小约35×28×22mm，T1WI低信号、T2WI高信号，内见分隔影，DWI呈高信号，增强后分隔及边缘明显强化，胰腺形态可，信号未见异常，增强后未见明显异常强化；肝脏形态，大小如常，血管纹理清晰，其内未见异常信号，增强扫描未见明显异常强化；门脉主干及大分支未见明显充盈缺损影；胆囊大小可，肝内外胆管无扩张；右肾见T1WI低信号、T2WI高信号影，径约4mm，增强后无强化，脾脏偏大，信号未见异常，左肾大小、形态、信号未见异常，增强后未见明显异常强化，后腹膜未见淋巴结增大。 结论：肝门区-胰颈上方团块病灶，左肝或尾叶来源囊腺瘤可能，脓肿不除外。 右肾小囊肿。 | 1. "右肾见T1WI低信号、T2WI高信号影，径约4mm，增强后无强化..." - Describe the right kidney lesion, but there is incomplete description | "径约4mm"modified to"最大径约4mm"or"横径约4mm" | Refuse, no need to modify |  |
| 发现：两侧额叶皮层下、放射冠见少许斑点状、斑片状的T1WI等低信号、T2WI及FLAIR高信号灶，弥散加权未见明显高信号；余脑实质信号未见异常。脑室形态可，脑池、脑沟未见明显异常，中线结构居中无移位。左侧上颌窦见囊状T1WI低信号、T2WI高信号影。 颈椎序列可，生理曲度变直，椎体边缘见轻度骨质增生，C5-6椎间隙略窄，余椎间隙无狭窄，C3/4、4/5、5/6、6/7椎间盘轻度突出，硬膜囊受压，所见脊髓内未见明显异常信号影。 腰椎大体可，生理曲度存在，椎体边缘见骨质增生，L5-S1椎体相对缘见条片状T1WI、T2WI高信号影，椎间隙无狭窄，L5/S1椎间盘见突出，T2WI信号减低，硬膜囊有受压，脊髓圆锥、终丝马尾形态及信号如常。 结论：脑内少许缺血灶。 C3/4、4/5、5/6、6/7椎间盘轻度突出，颈椎肥大。 L5/S1椎间盘突出伴变性。 腰椎骨质增生，L5-S1椎体终板炎。 | 1. "左侧上颌窦见囊状T1WI低信号、T2WI高信号影" - This discovery is not mentioned in the conclusion 2. "颈椎序列可...硬膜囊受压，所见脊髓内未见明显异常信号影" - The conclusion mentions"颈椎肥大"，but this phenomenon is not described in the findings | 1. Add maxillary sinus abnormalities to the conclusion  2. Delete the term "颈椎肥大" from the conclusion or add relevant descriptions in the discovery section | 1.Accept 2.Refuse, no need to modify |  |

**Supplementary Figure S1:** The proposed clinical workflow of local LLMs deployment.

**Supplementary Figure S2**: Example screenshot of integrating error detection into radiology reporting systems.

**References**

1.	Johnson, A.E., et al., *MIMIC-III, a freely accessible critical care database.* Sci Data, 2016. **3**: p. 160035.

2.	*The noteevents table*. 2015, MIMIC.
